# DOES CALORIC RESTRICTION SLOW THE PROCESS OF BIOLOGICAL AGING? EVIDENCE FROM THE CALERIE TRIAL

**DOI:** 10.1093/geroni/igac059.391

**Published:** 2022-12-20

**Authors:** Reem Waziry

**Affiliations:** Columbia University, New York, New York, United States

## Abstract

Calorie restriction (CR) slows aging and increases healthy lifespan in model organisms. We tested if CR slowed biological aging in humans using DNA methylation analysis of blood samples from N=197 participants in the Comprehensive Assessment of Long-term Effects ofReducing Intake of Energy (CALERIE™) randomized controlled trial. We quantified CR effects on biological aging by comparing change scores for six epigenetic-clock and Pace-of-Aging measures between n=128 CR-group and n=69 ad-libitum-control-group participants at 12- and24-month follow-ups. CR effects were strongest for DunedinPACE Pace of Aging (12-month Cohen’s d=0.3; 24-month Cohen’s d=0.2, p<0.01 for both), followed by DunedinPoAm and the GrimAge epigenetic clock, although effects for these measures were not statistically differentfrom zero (p>0.08). CR effects for other epigenetic clocks were in the opposite direction (all p>0.15). CALERIE intervention slowed Pace of Aging but showed minimal effect on epigenetic clocks hypothesized to reflect longer term accumulation of aging burden.

